# Anterior cingulate cortex γ-aminobutyric acid deficits in youth with depression

**DOI:** 10.1038/tp.2017.187

**Published:** 2017-08-22

**Authors:** V Gabbay, K A Bradley, X Mao, R Ostrover, G Kang, D C Shungu

**Affiliations:** 1 Department of Psychiatry, Icahn School of Medicine at Mount Sinai, New York, NY, USA; 2 Nathan S. Kline Institute for Psychiatric Research, Orangeburg, NY, USA; 3 Department of Radiology, Weill Cornell Medicine, New York, NY, USA

## Abstract

Abnormally low γ-aminobutyric acid (GABA) levels have been consistently reported in adults with major depressive disorder (MDD). Our group extended this finding to adolescents, and documented that GABA deficits were associated with anhedonia. Here we aimed to confirm our prior finding of decreased brain GABA in youth with depression and explore its associations with clinical variables. Forty-four psychotropic medication-free youth with MDD and 36 healthy control (HC) participants (12–21 years) were studied. Participants represent a combined sample of 39 newly recruited youth (MDD=24) and 41 youth from our previously reported study (MDD=20). GABA levels and the combined resonances of glutamate and glutamine (Glx) were measured *in vivo* in the anterior cingulate cortex using proton magnetic resonance spectroscopy. Youth with depression exhibited significantly lower GABA levels than HC in both the newly reported (*P*=0.003) and the combined (*P*=0.003) samples. When depressed participants were classified based on the presence of anhedonia, only the anhedonic MDD subgroup showed reduced GABA levels compared to HC (*P*=0.002). While there were no associations between any clinical measures and GABA or Glx levels in the new sample, GABA was negatively correlated with only anhedonia severity in the combined MDD group. Furthermore, in the combined sample, hierarchical regression models showed that anhedonia, but not depression severity, anxiety or suicidality, contributed significant variance in GABA levels. This report solidifies the evidence for a GABA deficit early in the course of MDD, which correlates specifically with anhedonia in the disorder.

## Introduction

Major depressive disorder (MDD) often emerges during adolescence, a critical developmental period in which many psychiatric conditions first develop.^1^ During adolescence, particularly after the initiation of puberty, the brain undergoes rapid maturation, a process that extends into the mid-twenties and may render youth more susceptible to the neural consequences of psychiatric illness.^2, 3^ Adolescent-onset MDD has severe consequences on many life domains, such as psychosocial functioning,^4^ and is most critically associated with suicide, the second leading cause of death in youth.^5^ Despite the importance of investigating neurobiological correlates of MDD early in the course of illness, most work has been done in adults. This study is an effort to address this gap and includes youth of ages 12–21 years old, in order to examine this crucial neurodevelopmental period.

Emerging evidence points to dysregulation of γ-aminobutyric acid (GABA), the primary inhibitory neurotransmitter, in MDD. Chief among such evidence is that GABA deficits have been consistently documented in adults with depression,^6, 7, 8, 9^ including those with treatment resistant depression.^10^ Furthermore, a recent mini-review of neuroimaging studies in adults with depression concluded that GABA levels might serve as a marker for illness state.^11^ However, only a few studies have specifically examined brain GABA levels in youth with depression^12^ or youth at risk for depression.^13^ Our laboratory extended findings of reduced GABA levels in adults with depression^6, 7, 8, 9^ to a younger cohort and reported decreased anterior cingulate cortex (ACC) GABA levels in adolescents with MDD.^12^ We further documented a negative association between GABA levels and anhedonia severity.^12^ Relatedly, several studies documented lower GABA levels in the cerebrospinal fluid (CSF),^14^ plasma^15^ and brain^8^ in adults with the melancholic subtype of depression, whose hallmark is severe anhedonia. Taken together, these findings point to the possible role of GABA deficits in MDD, and more specifically in anhedonic symptomatology. To our knowledge, to date, no additional studies have investigated brain GABA levels in youth with MDD.

Importantly, inconsistencies in biological research have impeded the development of reliable biomarkers for MDD, especially early in the course of illness, which has necessitated the replication of findings in both new and larger samples. Therefore, in the present study, we aimed to confirm our prior findings in a newly recruited sample of psychotropic medication-free youth with MDD and healthy controls (HCs), as well as in a large sample that combines our new and previously reported subjects.^12^ The latter would provide the largest GABA study in youth with MDD to date. On the basis of our previous investigation,^12^ our primary hypothesis was that youth with MDD would exhibit reduced ACC GABA compared to HC. Furthermore, when depressed participants were classified based on the presence of anhedonia, we expected only the anhedonic MDD subgroup to exhibit reduced GABA. In addition, we explored relationships between ACC GABA levels and clinical variables, including depression severity and episode duration, anhedonia, anxiety and suicidality. Furthermore, as alterations in the excitatory neurotransmitter glutamate have consistently been reported in mood disorders, including MDD,^16^ group differences in the combined resonances of glutamate and glutamine (Glx) were also explored; we expected no group differences in Glx in light of the negative findings regarding this neurometabolite in our prior study.^12^

## Materials and methods

### Participants

Twenty-four youth with MDD (mean age=16.07, s.d.=2.64; 46% female) and 15 HCs with no history of mental illness (mean age=15.33, s.d.=2.68; 53% female), ages 12–21 years (Tanner pubertal stage^17^ ⩾4), were newly recruited to our ongoing study (not newly diagnosed) and scanned; these participants are henceforth designated as the 'newly recruited sample'. Data from 20 youth with MDD and 21 HC, acquired as part of an earlier cohort for this same study using the same research-dedicated General Electric 3.0 Tesla Excite MRI scanner (GE Medical Systems, Milwaukee, WI, USA) and experimental protocol, were previously reported on,^12^ and combined with the newly recruited participants for a total sample of 44 youth with MDD (mean age=16.31, s.d.=2.64; 52% female) and 36 HC (mean age=15.80, s.d.=2.12; 64% female); this total sample is henceforth designated as the 'combined sample'. With our chosen sample of 80 participants, we have 80% power to detect moderate to large effects in group differences in GABA levels, which is sufficient given the large effects (f=0.5) found in our previous GABA study.^12^ Participants of 18 years of age or older provided informed consent, while those under 18 provided assent, with a parent or guardian providing informed consent. Institutional review boards at the Icahn School of Medicine at Mount Sinai, Weill Cornell Medicine, Nathan S. Kline Institute for Psychiatric Research, and New York University School of Medicine approved the study protocol.

Inclusion in the depressed group required meeting Diagnostic and Statistical Manual of Mental Disorders, Fourth Edition-Text Revision (DSM-IV-TR)^18^ criteria for MDD, with a current episode duration of at least 8 weeks, and a depression severity score of at least 35 on the Children’s Depression Rating Scale-Revised (CDRS-R).^19^ Comorbid diagnoses of attention-deficit/hyperactivity disorder (ADHD) and anxiety disorders were not exclusionary, to provide a generalized sample of adolescents with MDD. All participants with MDD were required to be psychotropic medication-free for at least seven half-lives of the medication. HC participants could not meet criteria for any current or past DSM-IV-TR disorder or have a history of treatment with psychotropic medication.

Exclusion criteria for all participants were an IQ below 80 on the Kaufman Brief Intelligence Test,^20^ the presence of significant medical, neurological or neurodevelopmental disorders, and contraindications to undergo magnetic resonance imaging (MRI). In addition, a positive urine toxicology test on the day of the scan, and in female participants, a positive pregnancy test, were exclusionary. Diagnoses of psychosis, bipolar disorder, pervasive development disorder, eating disorders or a current substance abuse disorder were exclusionary, as was acute suicidality requiring immediate medical attention.

### Clinical measures

#### Diagnoses

All participants were evaluated using the Kiddie-Schedule for Affective Disorders and Schizophrenia-Present and Lifetime version (KSADS). Even though the KSADS-PL is only validated for use in adolescents up to age 17,^21^ we used it for all participants, 18–21 years old, in order to maintain continuity of assessment and because the age range of adolescence is still rather vague and debated in psychiatric research. A board-certified child/adolescent psychiatrist or licensed clinical psychologist trained in administering the KSADS carried out the diagnostic evaluations, with the final clinical report discussed between the Primary Investigator (a licensed child/adolescent psychiatrist with expertise in adolescent depression) and the assessor to ensure diagnostic accuracy.

#### Depression severity

Depression severity was quantified using the CDRS-R. The CDRS-R is a reliable and validated semi-structured clinician-rated interview for the assessment of 17 depression symptom areas that distinguish depressed from non-depressed individuals.^19, 22, 23^ The score on the 'difficulty having fun' question, which relates to anhedonia, was subtracted from the final depression severity score. This was done in order to separately assess the contributions of overall illness severity and anhedonia severity, since this question was used in the calculation of our anhedonia measure described below.

#### Dimensional classification of anhedonia

Anhedonia severity was quantified by combining scores from two questions on the Beck Depression Inventory (BDI) and one question on the CDRS-R. The BDI is a heavily validated self-report depression severity measure with high internal consistency for psychiatric and non-psychiatric populations.^24, 25^ Specifically, scores from the 'loss of interest' and 'loss of pleasure' questions on the BDI (self-rated) and the 'difficulty having fun' question on the CDRS-R (clinician-rated) were summed. Self- and clinician-rated assessments contributed equally to the total combined anhedonia score; the two questions from the BDI are on a scale of 0–3, while the question from the CDRS-R is on a scale of 1–7. To ensure self- and clinician-rated items were on the same scale, one point was added to the sum of the two BDI questions before totaling the BDI and CDRS-R contributions. The total anhedonia severity scores thus ranged from 2 to 14. The Chronbach’s alpha for the three items (two BDI questions and one CDRS-R question) that make up this anhedonia metric was 0.83, demonstrating a high level of internal consistency for our measure. In addition, we have previously implemented this method of quantifying anhedonia,^12, 26, 27^ and found it to correlate strongly with a commonly used measure of anhedonia severity, the Snaith Hamilton Pleasure Scale (SHAPS).^28^ In a subset of participants from the current study (*n*=24; 19 MDD and 5 HC) that were also given the SHAPS, our anhedonia measure and the SHAPS were strongly correlated (*ρ*=0.80, *P*<0.0005), further validating its use in this study population.

#### Categorical classification of anhedonia

Depressed participants were additionally qualitatively classified as either anhedonic or non-anhedonic based on review of our anhedonia measure, as well as mood reactivity and clinician comments from the KSADS-PL interview. These designations were made by a child and adolescent psychiatrist without knowledge of the biological data. Anhedonic participants were required to be scored as having both anhedonia and lack of mood reactivity on the KSADS-PL.

#### Anxiety

Overall anxiety was quantified using the total score from the self-rated Multidimensional Anxiety Scale for Children (MASC),^29^ which has been validated with high internal consistency for use in the assessment of anxiety symptom severity.^29, 30^

#### Suicidality

The self-rated Beck Scale for Suicidal Ideation (BSSI), a scale with high internal consistency, was used for evaluation of suicidal ideation.^31^

### Structural MRI

All imaging data was collected on a 3.0 Tesla General Electric Excite HD MRI system (GE Medical Systems). A three-plane, low-resolution, high-speed scout imaging series was acquired, as well as standard high-resolution axial, coronal and sagittal T1-, T2- and spin density-weighted scans that were suitably obliqued for prescribing the ^1^H magnetic resonance spectroscopy (MRS) voxels of interest (VOI) in the ACC. In addition, a T1-weighted spoiled gradient-recalled echo (SPGR) volumetric scan and an axial Fast Fluid-Attenuated Inversion Recovery (FLAIR) scan were acquired for brain tissue segmentation and to rule out exclusionary focal brain lesions, respectively. All imaging parameters and procedures have been described previously.^12^

### ^1^H MRS

**Figure 1:**
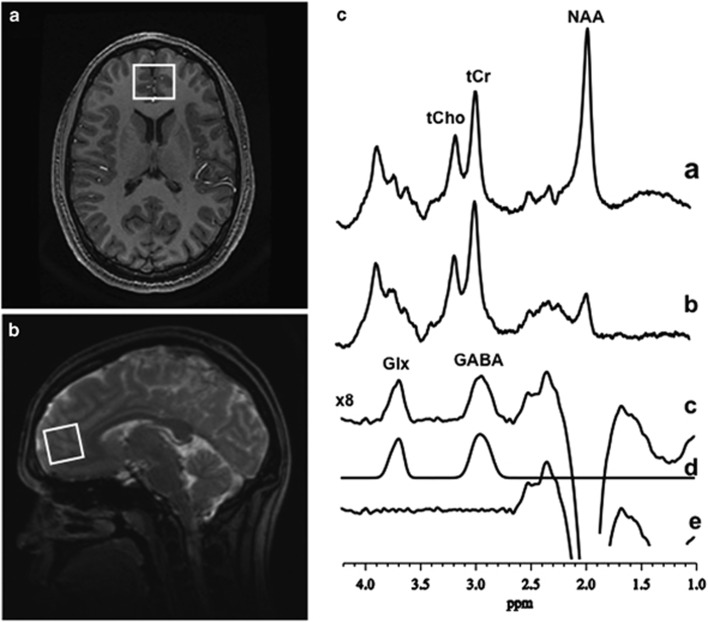
Proton magnetic resonance spectroscopy (^1^H MRS) examples voxel placement and spectra. (**a**) Axial and (**b**) sagittal localizer images showing the size and location of the anterior cingulate cortex (ACC) voxel of interest. (**c**) Demonstration of *in vivo* human GABA and Glx detection by ^1^H MRS in the voxel of interest: (a and b), single-voxel subspectra acquired in 15 min with the editing pulse on and off and 290 (580 total) interleaved averages; spectrum (c), difference between spectra (a and b) showing the edited brain GABA and Glx resonances; spectrum (d), model-fitting of the experimental spectrum (**c**) to obtain the GABA and Glx peak areas; spectrum (e), residual of the difference between spectra (c and d). GABA, γ-aminobutyric acid; Glx, glutamate+glutamine; NAA, N-acetyl-aspartate; tCho, total choline; tCr, total creatine.

A standard J-edited spin echo difference method^32^ was used to acquire the GABA-edited ^1^H MRS data. Data were processed as demonstrated in Figure 1 and as fully described previously.^12, 33^ Each spectrum was recorded from single 2.5 × 2.5 × 3.0 cm^3^ voxels prescribed in the ACC (Figure 1). Details of the MRS data quality assessment criteria and procedures used to retain or reject spectra are provided in Shungu *et al.*^33^ Spectral peak areas, which are proportional to the concentrations of the associated metabolites, were obtained for cases that fulfilled quality assessment criteria (Figure 1). The GABA and Glx resonances and co-edits within the J-edited difference spectra were modeled as a linear combination of pseudo-Voigt lineshape functions and fitted in the frequency domain using a robust and highly optimized public-domain Levenberg–Marquardt nonlinear least-squares minimization routine.^34^ The GABA and Glx levels were expressed as ratios of peak areas relative to the area of the simultaneously acquired and similarly fitted unsuppressed voxel water signal (W) for normalization across subjects (that is, GABA/W and Glx/W).

### Brain tissue segmentation

MEDx software (Medical Numerics, Germantown, MD, USA) was used to segment brain tissue and estimate the proportions of gray matter (GM), white matter (WM) and CSF in each VOI based on the signal-intensity histogram of each participant’s volumetric (SPGR) MRI. A segmentation mask for each voxel was created using in-house software developed in MATLAB (MathWorks, Natick, MA, USA); the proportions of GM, WM and CSF were determined from these segmentation masks and were compared between groups. Any tissue variables showing significant relations to GABA and Glx were included as covariates in analysis of covariance (ANCOVA) models that compared groups on these metrics.

### Statistical analyses

All statistical analyses were conducted using SPSS Statistics, version 23 (IBM, Armonk, NY, USA). Descriptive statistics were conducted, and normality of data was assessed using the Shapiro-Wilk test in order to evaluate assumptions for all statistical analyses. Groups (that is, HC and MDD) were compared on all demographic and clinical variables using independent samples *t*-tests; when parametric assumptions were not met, nonparametric test equivalents were utilized (for example, Mann–Whitney *U*-Test). Group differences in voxel tissue heterogeneity (that is, W, GM, WM and CSF) were also specifically assessed using ANCOVA, controlling for age and sex, given that these factors have been found to influence brain tissue makeup.^35^

To test our primary hypothesis that youth with depression exhibit lower ACC GABA levels than HCs, groups (that is, MDD and HC) were compared using ANCOVA in the newly recruited sample (24 MDD, 15 HC), as well as in the combined sample (44 MDD, 36 HC). In addition, GABA levels were compared between anhedonic and non-anhedonic MDD subgroups and HCs, in the combined sample only; the sample size of the newly recruited cohort was too small to conduct these analyses. ANCOVAs were similarly used to examine these subgroup differences. A Sidak correction for multiple comparisons was applied to the three-group comparison with α=0.05. For all ANCOVAs, variables known to be related to GABA were examined as covariates in the statistical models; relationships between the neurochemicals and age, sex, ethnicity, as well as GM%, WM% and CSF% in the VOI were examined. Only variables that were significantly correlated (*P*<0.05), approached a significant correlation (*P*<0.10) or showed differences in the neurochemicals were included as covariates in the ANCOVA models that compared GABA between groups. Similar ANCOVAs were used to explore differences in Glx levels between MDD and HC groups in the newly recruited and combined samples.

Pearson’s ('r') or Spearman’s ('rho' or '*ρ*') correlations (two-tailed) were used to assess relationships between clinical variables (that is, depression severity and duration, anhedonia, anxiety and suicidality) and neurochemicals (that is, GABA/W and Glx/W) in the full MDD group alone and the full participant group (HC+MDD) of the newly recruited and combined samples. We include correlations in both the full MDD group alone and the full participant group (HC+MDD), as symptoms such as anxiety and anhedonia do exist in some level in HCs, even if not clinically significant. Therefore, in line with a Research Domain Criteria (RDoC) approach, whereby behavioral constructs are evaluated along continuums,^36, 37^ we conduct these dimensional analyses in both 'ill' and full participant (HC+MDD) groups. The type of correlation coefficient used was based on whether parametric (Pearson’s) or nonparametric (Spearman’s) assumptions were met. Partial Pearson’s or Spearman’s rank-order correlations between anhedonia and neurochemicals controlled for depression severity, while partial correlations between depression severity and neurochemicals controlled for anhedonia.

Finally, hierarchical multiple linear regression was conducted in the combined MDD sample to assess whether anhedonia predicts unique and significant variance in GABA in youth with MDD, separate from anxiety, suicidality and depression severity. The first model included anxiety. The second model added suicidality. The third model added anhedonia, and the fourth model added depression severity. In order to exclude order effects between anhedonia and depression severity, the hierarchical regression was repeated with the third and fourth models reversed so that anhedonia was input last.

All analyses were conducted with an *α*=0.05.

## Results

### Demographics

Table 1 summarizes the demographical characteristics of all participants. There were no significant differences between the HC and MDD groups in either the newly recruited or combined samples on any demographic variables, except for ethnicity in the combined sample (*X*^2^=8.42, *P*=0.04).

There were no significant correlations between age and GABA/W or Glx/W in all participants (HC+MDD) from either the newly recruited (24 MDD, 15 HC) or combined (44 MDD, 36 HC) samples (all *P*>0.10). However, in the MDD group alone (*n*=44) of the combined sample, there was a negative correlation between GABA/W and age (ρ=−0.32, *P*=0.03). In addition, there were no differences in neurometabolites based on ethnicity (all *P*>0.10). Moreover, in the combined sample, there were neurochemical differences between the sexes. Glx/W was higher in male (2.08 × 10^−3^ (4.36 × 10^−4^)) than female (1.87 × 10^−3^ (4.55 × 10^−4^)) participants (t(78)=2.11, *P*=0.04, 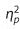
=0.05), but there were no sex differences in GABA/W. Given that age and sex may be related to neurometabolite levels to some degree, these variables were included as covariates in all ANCOVA models. In addition, even though there was no relationship between ethnicity and neurometabolite levels, this variable was significantly different between MDD and HC groups and was thus also included as a covariate.

### Clinical characteristics

Clinical characteristics of the sample are also provided in Table 1. All depressed participants were medication-naive (*n*=32) or medication-free (*n*=11), except for one participant who began taking an antidepressant (Sertraline) 2 days prior to the scan. The data for this participant were nevertheless included due to the brief treatment duration. Analyses were conducted both with and without these data.

#### Anhedonia and depression severity

Spearman’s correlations assessed the relationship between anhedonia and depression severity. In the newly recruited sample (24 MDD, 15 HC), anhedonia and depression severity were correlated in the MDD group (*ρ*=0.58, *P*<0.003) and the full participant group (HC+MDD) (*ρ*=0.82, *P*<0.0005). Similarly, in the combined sample (44 MDD, 36 HC), anhedonia and depression severity were also correlated in the MDD group (*ρ*=0.58, *P*<0.0005) and the full participant group (*ρ*=0.85, *P*<0.0005).

### Voxel tissue heterogeneity

In both the newly recruited (24 MDD, 15 HC) and the combined (44 MDD, 36 HC) samples, there were no significant differences between groups (that is, MDD, HC) in the levels of unsuppressed voxel tissue water signal (W) (newly recruited: F(1,35)=1.09, *P*=0.30, 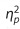
=0.03; combined: F(1,76)=1.03, *P*=0.31, 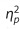
=0.01). Therefore, ratios of GABA/W and Glx/W were used in all analyses, which will be referred to henceforth simply as GABA and Glx, respectively. In addition, in the newly recruited sample, there were no differences in GM% (F(1,34)=0.98, *P*=0.33, 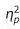
=0.03), WM% (F(1,34)=1.41, *P*=0.24, 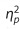
=0.04) or CSF% (F(1,34)=1.50, *P*=0.23, 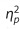
=0.04) or correlations between neurometabolites and voxel tissue variables (all *P*>0.10). Similarly, in the combined sample, there were no significant differences between groups in GM% (F(1,75)=1.14, *P*=0.29, 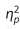
=0.02) or WM% (F(1,75)=2.58, *P*=0.11, 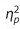
=0.03), and there were no correlations between GABA or Glx and these variables (all *P*>0.10). However, differences between groups in mean CSF% approached significance (F(1,75)=3.22, *P*=0.08, 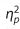
=0.04), and while GABA did not correlate with CSF%, there was a trend-level correlation between Glx and CSF% (*r*=−0.21, *P*=0.06). Therefore, CSF% was included as a covariate in all ANCOVA models that compared mean neurochemical levels between groups. All voxel tissue values (that is, W, GM, WM and CSF) for the newly recruited and combined samples are presented in Table 2.

### Primary hypothesis

#### GABA group differences

**Figure 2:**
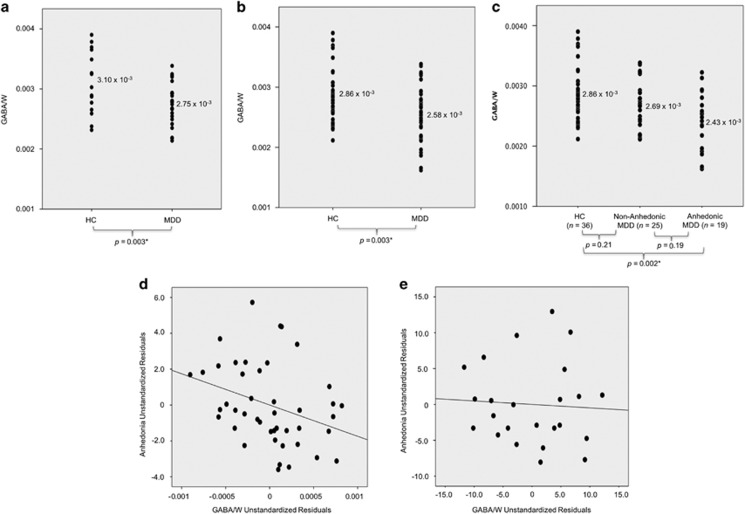
GABA/W group differences and clinical correlations. Analysis of covariance (ANCOVA), controlling for age, sex, ethnicity and cerebrospinal fluid in the voxel of interest compared GABA levels between groups (α=0.05). (**a**) Newly recruited sample: GABA/W in MDD and HC. GABA/W was significantly lower (*P*=0.003) in MDD compared to HC. (**b**) Combined sample: GABA/W in MDD and HC. GABA/W was significantly lower (*P*=0.003) in MDD compared to HC. (**c**) Combined sample: GABA/W in anhedonic MDD, non-anhedonic MDD, and HC. A Sidak correction for multiple comparisons was utilized for the three-group comparison. GABA/W was significantly lower in the anhedonic MDD subgroup compared to HC (*P*=0.002). Differences between the MDD subgroups (*P*=0.19), as well as between the non-anhedonic MDD subgroup and HC were not significant (*P*=0.21). (**d**) Combined sample: partial correlation between GABA/W and anhedonia (controlling for depression severity) in MDD (*r*=−0.33, *P*=0.03) was significant (44 MDD). (**e**) Newly recruited sample: partial correlation between GABA/W and anhedonia (controlling for depression severity) in MDD (*r*=−0.06, *P*=0.79) was not significant (24 MDD).

*MDD* *vs* *HC:* As hypothesized, ANCOVAs, controlling for age, sex, ethnicity and CSF% in the VOI, revealed decreased GABA levels in youth with MDD compared to HC in both the newly recruited (F(1,32)=10.21, *P*=0.003, 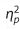
=0.24) and combined samples (F(1, 73)=9.21, *P*=0.003, 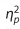
=0.11) (Table 2 and Figure 2). Levene’s test of equality of error variances was not significant in the newly recruited (*P*=0.51), or combined (*P*=0.88) samples, suggesting variance between the groups was not significantly different in either sample. In addition, without controlling for any variables, results of ANOVAs remained unchanged in both the newly recruited (F(1, 37)=6.64, *P*=0.01, 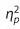
=0.15) and combined samples (F(1,78)=8.13, *P*=0.006, 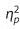
=0.09), with GABA levels decreased in youth with MDD compared to HC.

*Anhedonic MDD* *vs* *non-anhedonic MDD* *vs* *HC:* In the combined sample, GABA levels were different between the anhedonic and non-anhedonic MDD subgroups and HC (F(2, 72)=6.47, *P*=0.003, 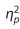
=0.15) when controlling for age, sex, ethnicity and CSF% in the VOI (Supplementary Table S2 and Figure 2). Follow-up pairwise comparisons revealed lower GABA levels in anhedonic youth with MDD compared to HC (*P*=0.002). However, GABA levels did not differ between the anhedonic and non-anhedonic MDD subgroups (*P*=0.19) or between the non-anhedonic MDD and HC groups (*P*=0.21). Results remained unchanged when not controlling for any variables in the model (F(2, 77)=6.15, *P*=0.003, 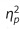
=0.14; anhedonic MDD vs HC *P*=0.002; anhedonic MDD vs non-anhedonic MDD *P*=0.15; non-anhedonic MDD vs HC *P*=0.35).

### Exploratory analyses

#### Dimensional analyses between GABA and clinical measures

*Newly recruited sample:* No associations between GABA and clinical variables (that is, anhedonia, depression severity, anxiety and suicidality) were documented in the newly recruited MDD group alone or the newly recruited full participant group (HC+MDD; all *P*>0.05). In addition, there were no correlations between GABA and MDD episode duration in the newly recruited MDD group alone (*ρ*=0.19, *P*=0.40).

*Combined sample:* GABA was negatively associated with anhedonia in the MDD group alone (*r*=−0.33, *P*=0.03) (Figure 2). However, in the full participant group (HC+MDD), the negative association between GABA and anhedonia only approached significance (*ρ*=−0.21, *P*=0.08). No other correlations were documented between GABA and any other clinical variables (that is, depression severity, anxiety and suicidality) in either the MDD group alone or the full participant group (HC+MDD; all *P*>0.05). In addition, GABA was not correlated with MDD episode duration in the combined MDD group alone (*ρ*=0.08, *P*=0.63).

#### Hierarchical multiple regression in MDD

Hierarchical multiple regression was only conducted in the combined MDD sample (44 MDD) due to power considerations. The full model (that is, anxiety, suicidality, anhedonia and depression) accounted for 15.90% of the variance in GABA levels in the MDD group (Table 3). Importantly, anhedonia (*β*=−0.43, *P*=0.02), but not depression severity (*β*=−0.01, *P*=0.96), anxiety (*β*=−0.14, *P*=0.38) or suicidality (*β*=−0.06, *P*=0.70), significantly predicted GABA in MDD. Anhedonia predicted 13.50% of the variance in GABA, while depression severity accounted for <0.05%, anxiety for 2.00% and suicidality for 0.40%.

To exclude the effects of anhedonia order in the hierarchical models, the same models were repeated, but with anhedonia entered as the last variable in the model. These results remained unchanged, with anhedonia as the only significant predictor of variance in GABA levels (Supplementary Table S1).

### Additional GABA MDD subgroup differences

#### Single episode MDD vs recurrent MDD

In the combined sample, GABA levels were significantly lower in depressed individuals with recurrent episodes compared to single episodes (F(1, 41)=16.41, *P*<0.0005, 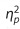
=0.29) when controlling for age, sex, ethnicity and CSF% in the VOI. Results remained unchanged when not controlling for any variables (F(1, 41)=16.41, *P*<0.0005, 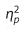
=0.29). Analyses were not done in the newly recruited MDD sample due to the small sample size (that is, only seven participants with recurrent depressive episodes).

#### Glx group differences and clinical correlations

No differences in Glx levels were found between the MDD and HC groups in either the newly recruited (F(1, 32)=0.022, *P*=0.88, 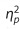
=0.001) or combined samples (F(1, 73)=0.009, *P*=0.93, 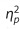
<0.0005) when controlling for age, sex, ethnicity and CSF% in the VOI (Table 2) or when not controlling for any variables (newly recruited sample: F(1, 37) <0.0005, *P*=1, 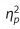
<0.0005; combined sample: F(1, 78)=0.002, *P*=0.97, 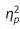
<0.0005).

In addition, in the combined MDD sample, Glx levels were not different between individuals with single or recurrent depressive episodes (F(1, 41)=0.332, *P*=0.57, =0.008) when controlling for age, sex, ethnicity and CSF% in the VOI, or when not controlling for anything (F(1, 41)=0.332, *P*=0.57, =0.008).

No correlations were found between Glx and any clinical variables (that is, anhedonia, depression severity, anxiety and suicidality) in the MDD group alone or the full participant group (HC+MDD) of either the newly recruited or combined samples (all *P*>0.05). Glx was also not correlated with MDD episode duration (newly recruited sample: *ρ*=−0.08, *P*=0.72; combined sample: *ρ*=−0.19, *P*=0.24).

All primary hypothesis testing and exploratory results remained unchanged when excluding the one medicated participant with MDD.

## Discussion

This study has confirmed our prior finding of decreased ACC GABA levels in youth with MDD.^12^ We found that youth with depression had significantly lower ACC GABA levels compared to HC in both the newly recruited and combined samples. In the combined sample, this decrease was related to anhedonia severity. Specifically, when patients were classified based on the presence of anhedonia, only the anhedonic MDD subgroup exhibited reduced GABA compared to HC. In addition, we documented a negative correlation between anhedonia severity and GABA levels in the combined MDD group, even when controlling for depression severity. The hierarchical multiple linear regression models further suggested that significant variance in GABA was attributable to anhedonia, rather than any other clinical symptom, including overall depression severity, anxiety and suicidality. Interestingly, we also found that GABA levels were significantly reduced in depressed individuals who suffered recurrent episodes compared to those who only experienced a single episode. Finally, as in our prior study, exploratory analyses of the present data found no group differences in Glx or any relationships between Glx and clinical symptomatology. Collectively, these results suggest an early presence of GABA deficits in youth with depression and a corresponding relation to both anhedonia severity and more severe recurrent illness.

Not only is our broad finding of decreased GABA in youth with depression consistent with our previous smaller sample investigation of adolescents with MDD,^12^ but it is also in line with other ^1^H MRS studies of actively^6, 7, 8, 9, 10^ and remitted^38^ depressed adults. Moreover, GABA deficits have been found in the plasma^15, 39^ and CSF^14, 40, 41^ of adults with depression.^42^ Postmortem investigations further support these findings and have shown decreased density of GABAergic interneurons in the auditory cortex of adults with depression compared to non-psychiatric controls,^43^ as well as small size and low density of GABAergic interneurons immunoreactive for the calbindin protein in the dorsolateral prefrontal cortex (DLPFC) of adults with depression.^44^ Glutamic acid decarboxylase (GAD) protein, a GABA synthesizing enzyme, is also reduced in the DLPFC of adults with depression.^45^ The similarities of these findings with those of the current investigation add weight to the evidence implicating dysfunction of the GABAergic system in major depression.

Another crucial finding in the present study is the association between ACC GABA levels and anhedonia. While there was no correlation between GABA levels and anhedonia in the newly recruited sample, there was a strong negative correlation in the combined MDD group. One possible explanation for this result is the smaller sample size and restricted range of anhedonia severity in the newly recruited cohort of youth with depression (that is, fewer participants with anhedonia severity >8 in the newly recruited MDD sample; Supplementary Figure S1). Furthermore, we also found that only the anhedonic MDD subgroup exhibited lower GABA compared to HC. These results are consistent with our prior ^1^H MRS study of GABA in adolescent MDD,^12^ as well as other ^1^H MRS^8^ and CSF concentration^14^ studies of GABA in adult melancholic MDD, of which anhedonia is a hallmark characteristic. Moreover, we also recently documented that anhedonia in adolescent MDD is associated with more severe outcomes, including suicidality, episode duration and number of episodes.^28^ Importantly, Price *et al.*^10^ documented that GABA levels were specifically decreased in a group of patients with treatment resistant depression, also characterized by more severe outcomes. Here we also found that depressed individuals who experienced multiple depressive episodes exhibited reduced GABA levels compared to those that only experienced a single episode. Collectively, these results suggest that GABA deficits might be associated with either anhedonia specifically or with a poorer, more severe outcome in general in MDD.

Since anhedonia reflects deficits of reward processing,^46, 47^ our findings may be explained by the regulatory role of GABA in neuronal reward processing. Alterations in the GABAergic system frequently occur in prefrontal cortical regions such as the ACC, as evidenced by our laboratory’s investigations,^12^ as well as in other reward-related regions.^48^ The reward circuitry is primarily comprised of the prefrontal cortex, nucleus accumbens, ventral tegmental area, habenula, striatum, hypothalamus, amygdala and hippocampus.^47^ One theory is that deficits of GABA may induce alterations within the dopamine-mediated reward circuitry. Animal studies have specifically shown that GABA modulates dopamine in the midbrain tegmental area and striatum, key reward regions connected to the ACC.^49, 50^ Moreover, more recent multimodal investigations have shown a link between GABA levels and both resting-state functional connectivity and task-based activity in reward-related regions, further implicating the regulatory role of GABA in reward processing.^51^ Therefore, alterations in the GABAergic system may lead to disruptions in reward circuitry due to its influence on dopamine regulation. Nonetheless, while our study focused on the ACC, it is not clear if our finding is specific to the prefrontal cortex, as others have also found decreased GABA in MDD in other brain regions, including in the occipital cortex.^52^ More research is thus necessary to assess GABA levels across different cortical and subcortical (for example, striatum) brain regions.

Although the primary focus of the current investigation was on GABA, this inhibitory neurotransmitter system is tightly linked to the excitatory system of glutamate, which is the most abundant neurotransmitter in the central nervous system and a synthetic precursor for both glutamine and GABA.^16^ While both our current and prior studies^12^ did not find group differences in Glx or associations between Glx and MDD symptoms, there have been reports linking disturbances in the glutamatergic system with MDD, both in animals^53^ and humans.^16^ One important caveat that may explain the discrepancy between our lack of group differences in Glx levels and the current literature is the age range of our MDD sample. It is possible that in our sample of youth with depression, alterations in Glx metabolism are not yet apparent due to shorter disease chronicity, whereas many previous studies of Glx have been conducted in adults.^16^

Several methodological limitations should be noted. First, recruitment of a broad age range (12–21 years old) may have neuroendocrine consequences. Even though participants who met Tanner pubertal stage 4 or greater criteria were recruited, there may still be endocrine variation within the sample that is not accounted for, which could impact the results. In addition, given the single clinician procedure for clinical evaluation of the participants in this study, kappas could not be calculated for inter-rater reliability of the KSADS-PL or the CDRS-R. However, these evaluations were conducted by a board-certified child/adolescent psychiatrist or licensed clinical psychologist trained in administering these assessments and reviewed by a licensed child and adolescent psychiatrist specializing in pediatric depression. Moreover, the KSADS-PL was used for all participants ages 12–21 years for consistency in evaluation, despite that it is only validated for use in adolescents through age 17.^21^ Moreover, the ^1^H MRS sequence used in our investigation did not allow for the separate assessment of glutamate and glutamine resonances, thus only allowing the quantification of combined resonances of the two (that is, Glx). Furthermore, the GABA sequence contained up to a 50% contribution from mobile macromolecules. Similarly, our voxel size was considerably large, although consistent with other 3.0 Tesla ^1^H MRS studies. Despite these methodological limitations, the use of a larger, medication-free sample of youth with depression, most of which was psychotropic medication-naive, precludes the effects of medications and disease chronicity, two factors that frequently impede the assessment of biomarkers of MDD in psychiatric research.

In conclusion, this study suggests that GABAergic system dysfunction plays a role early in the course of MDD. Furthermore, given that GABA alterations were present in the ACC, a region important for reward processing^47^ and related specifically to anhedonia severity in our current and previous investigations,^12^ these findings may indicate that reward circuitry dysfunction underlies the pathophysiology of anhedonia in adolescent depression. However, anhedonia is present in many psychiatric disorders and is a prodromal symptom for future psychopathology, and thus future longitudinal studies should assess the neurobiology of reward system dysfunction in more diverse samples of adolescents with psychopathology in order to better understand the mechanisms underlying this behavioral construct. Such work aligns with the goals of the NIMH’s RDoC,^36, 37^ which seeks to explore the underlying neurobiology of specific behavioral constructs that cut across psychiatric diagnoses to facilitate the identification of biomarkers for illness treatment and progression.

## Figures and Tables

**Table 1:** Demographic and clinical characteristics of the study samples

*Demographic variables*	*Newly recruited sample*			*Combined sample*		
	*MDD (*n=*24)*	*HC (*n=*15)*	P*-value*	*MDD (*n=*44)*	*HC (*n=*36)*	P*-value*
Age (mean±s.d.)	16.07±2.64	15.33±2.68	0.41	16.31±2.64	15.80±2.12	0.33
Sex *(n*, female) (%)	11 (46%)	8 (53%)	0.65	23 (52%)	23 (64%)	0.30
						
*Ethnicity (*n*) (%)*			0.74			0.04^a^
Caucasian	9 (38%)	4 (27%)		17 (39%)	13 (36%)	
African American	7 (29%)	7 (47%)		9 (20%)	14 (39%)	
Hispanic	6 (25%)	3 (20%)		14 (32%)	3 (8%)	
Asian/other	2 (8%)	1 (7%)		4 (10%)	6 (16%)	
						
*Clinical variables (mean±s.d.) (range)*						
Anhedonia	6.58±2.65 (2–12)	2.30±0.67 (2–4)	<0.0005^a^	7.45±2.77 (2–12)	2.33±0.64 (0–4)	<0.0005^a^
CDRS-R	46.71±11.21 (35–79)	18.80±2.74 (17–25)	<0.0005^a^	49.84±10.13 (35–79)	18.80±1.94 (17–25)	<0.0005^a^
BDI-II	20.17±11.86 (0–40)	3.00±3.69 (0–10)	<0.0005^a^	24.32±12.76 (0–51)	3.00±3.34 (0–10)	<0.0005^a^
BSSI	4.21±6.12 (0–21)	0±0(0–0)	<0.0005^a^	4.53±6.20 (0–26)	.08±0.28 (0–1)	<0.0005^a^
MASC total	44.86±16.70 (2–76)	28.86±15.34 (4–57)	0.007^a^	48.10±22.11 (0–89)	28.00±14.32 (4–57)	<0.0005^a^
						
*Illness history*						
*Current comorbidity (*n*) (%)*						
ADHD	6 (25)	0 (0)		7 (16)	0 (0)	
Generalized anxiety disorder	8 (33)	0 (0)		15 (34)	0 (0)	
Separation anxiety disorder	1 (4)	0 (0)		1 (2)	0 (0)	
Specific phobia	1 (4)	0 (0)		1 (2)	0 (0)	
Social anxiety disorder	4 (17)	0 (0)		5 (11)	0 (0)	
OCD	1 (4)	0 (0)		1 (2)	0 (0)	
PTSD	1 (4)	0 (0)		1 (2)	0 (0)	
						
*Number of episodes (*n*) (%)*						
Single	16 (67)	0 (0)		28 (64)	0 (0)	
Recurrent	7 (29)	0 (0)		15 (34)	0 (0)	
						
Episode duration in months (mean±s.d.) (range)^b^	48.36±49.02 (8–208)	0 (0)		53.43±51.18 (8–208)	0 (0)	

Abbreviations: ADHD, attention-deficit/hyperactivity disorder; BDI-II, Beck Depression Inventory, Second Edition; BSSI, Beck Scale of Suicidal Ideation; CDRS-R, Children’s Depression Rating Scale-Revised; HC, healthy controls; MASC, Multidimensional Anxiety Scale for Children; MDD, major depressive disorder; OCD, obsessive-compulsive disorder; PTSD, post-traumatic stress disorder.

a Significant difference between the MDD and HC groups (*P*<0.05).

b A Mann–Whitney *U-*Test revealed no difference in MDD episode duration between the newly recruited depressed participants and the combined sample (*U*=205.5, *P*=0.71).

**Table 2:** Anterior cingulate cortex voxel tissue heterogeneity variables, GABA/W levels, and Glx/W levels

	*Newly recruited sample*					*Combined sample*				
	*MDD (*n=*24)*		*HC (*n=*15)*			*MDD (*n=*44)*		*HC (*n=*36)*		
	*Mean*	*s.d.*	*Mean*	*s.d.*	P*-value*	*Mean*	*s.d.*	*Mean*	*s.d.*	P*-value*
GM %	5.80 × 10^−^^1^	2.55 × 10^−2^	5.69 × 10^−^^1^	3.52 × 10^−^^2^	0.33	5.83 × 10^−^^1^	3.26 × 10^−^^2^	5.71 × 10^−^^1^	4.16 × 10^−2^	0.29
WM %	2.91 × 10^−1^	3.05 × 10^−2^	3.06 × 10^−1^	4.65 × 10^−2^	0.24	2.88 × 10^−1^	3.44 × 10^−2^	3.03 × 10^−1^	4.32 × 10^−2^	0.11
CSF %	1.24 × 10^−1^	9.78 × 10^−3^	1.19 × 10^−1^	2.26 × 10^−2^	0.23	1.25 × 10^−1^	1.58 × 10^−2^	1.18 × 10^−1^	2.43 × 10^−2^	0.08^a^
W %	1.61 × 10^12^	4.98 × 10^11^	1.79 × 10^12^	5.22 × 10^11^	0.30	1.60 × 10^12^	4.41 × 10^11^	1.71 × 10^12^	4.16 × 10^11^	0.31
GABA/W	2.75 × 10^−3^	3.5 × 10^−4^	3.10 × 10^−3^	5.2 × 10^−4^	0.003^b^	2.58 × 10^−3^	4.4 × 10^−4^	2.86 × 10^−3^	4.4 × 10^−4^	0.003^b^
Glx/W	2.05 × 10^−3^	3.5 × 10^−4^	2.05 × 10^−3^	5.7 × 10^−4^	0.88	1.96 × 10^−3^	4.1 × 10^−4^	1.96 × 10^−3^	5.1 × 10^−4^	0.93

Abbreviations: CSF, cerebrospinal fluid; GABA, γ-aminobutyric acid; Glx, glutamate+glutamine; GM, gray matter; HC, healthy controls; MDD, major depressive disorder; W, unsuppressed voxel tissue water signal; WM, white matter.

a Group difference (MDD vs HC) approached significance (*P*<0.10).

b Significant difference between MDD and HC groups (*P*<0.05).

**Table 3:** Summary of hierarchical regression results: variables predicting GABA/W

*Variables*	*B*	*B-SE*	*Beta*	t	P*-value*	R	R^*2*^	*Δ*R^*2*^	*Sig. ΔF*
*Model 1*						0.14	0.02	0.02	0.38
Anxiety	−3.13 × 10^−6^	<0.0005	−0.14	−0.88	0.38				
*Model 2*						0.16	0.02	0.004	0.70
Suicidality	−4.52 × 10^−6^	<0.0005	−0.06	−0.39	0.70				
*Model 3*						0.40	0.16	0.14	0.02*
Anhedonia	−6.87 × 10^−5^	<0.0005	−0.43	−2.40	0.02*				
*Model 4*						0.40	0.16	<0.0005	0.96
Depression	−4.87 × 10^−7^	<0.0005	−0.01	−0.05	0.96				

Abbreviations: GABA, γ-aminobutyric acid; W, unsuppressed voxel tissue water signal.

*Significant (*P*<0.05).
