# Supplementary material for: Anterior cingulate cortex γ-aminobutyric acid deficits in youth with depression
Source: Transl Psychiatry. 2017 Aug 22;7(8):e1216–. doi: 10.1038/tp.2017.187 (PMC5611750; doi:10.1038/tp.2017.187)
Supplement: Supplementary Information [file tp2017187x1.docx]

**Supplemental Figure Legends**

**Figure S1. Anhedonia Distributions.** Anhedonia distributions in the newly recruited (24 MDD, 15 HC) and combined samples (44 MDD, 36 HC). MDD = major depressive disorder; HC = healthy control.

**Table S1. Summary of Hierarchical Regression Results: Variables Predicting GABA/W, with Anhedonia and Depression Severity Order Reversed**

| **Variables** | **B** | **B-SE** | **Beta** | **t** | **p** | **R** | **R^2^** | **ΔR^2^** | **Sig. ΔF** |
| --- | --- | --- | --- | --- | --- | --- | --- | --- | --- |
| **Model 1** |  |  |  |  |  | .14 | .02 | .02 | .38 |
| Anxiety | -3.13x10^-6^ | <.0005 | -.14 | -.88 | .38 |  |  |  |  |
| **Model 2** |  |  |  |  |  | .16 | .02 | .004 | .70 |
| Suicidality | -4.52x10^-6^ | <.0005 | -.06 | -.39 | .70 |  |  |  |  |
| **Model 3** |  |  |  |  |  | .24 | .06 | .03 | .26 |
| Depression | -9.50 x10^-6^ | <.0005 | -.20 | -1.14 | .26 |  |  |  |  |
| **Model 4** |  |  |  |  |  | .40 | .16 | .10 | .05* |
| Anhedonia | -6.78x10^-5^ | <.0005 | -.43 | -2.05 | .048* |  |  |  |  |

*significant (*p* < .05)

**Table S2: GABA/W Levels in Anhedonic MDD Subgroups and HC**

| **Variables [mean ± SD]** | **Anhedonic**  **MDD**  **(*n* = 19)** | **Non-Anhedonic**  **MDD**  **(*n* = 25)** | **HC**  **(*n* = 36)** |
| --- | --- | --- | --- |
| GABA/W | 2.43 x 10^-3^ ± 4.71 x 10^-4^* | 2.69 x 10^-3^ ± 3.88 x 10^-4^ | 2.86 x 10^-3^ ± 4.39 x 10^-4^* |

Abbreviations: W = unsuppressed voxel tissue water signal; GABA = gamma-aminobutyric acid; SD = standard deviation.

*The anhedonic MDD and HC groups were significantly different (*p* = .002)
