# Supplementary figures and images for: Anterior cingulate cortex γ-aminobutyric acid deficits in youth with depression
Source: Transl Psychiatry. 2017 Aug 22;7(8):e1216–. doi: 10.1038/tp.2017.187 (PMC5611750; doi:10.1038/tp.2017.187)

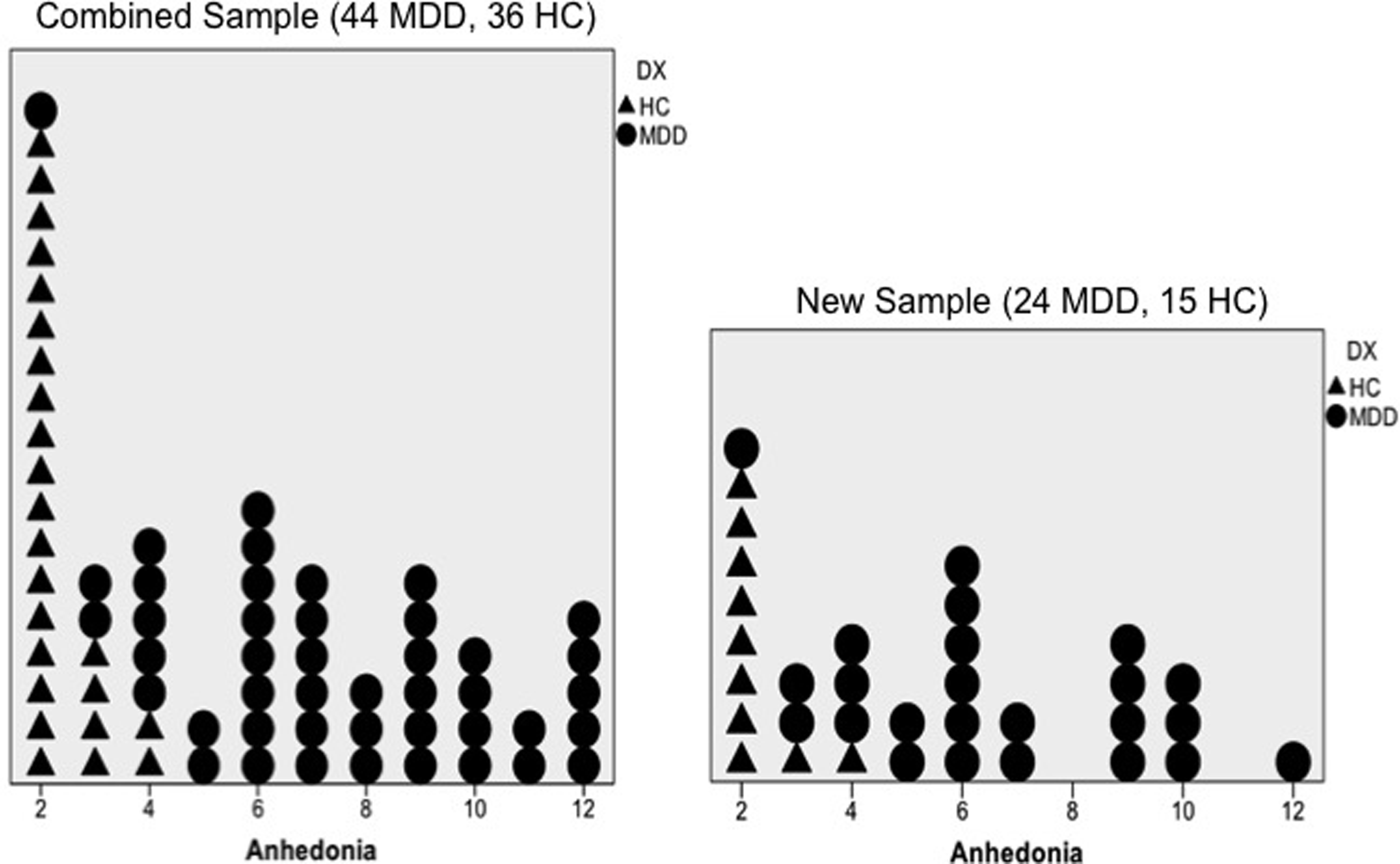

Supplement: Supplementary Figure 1 [file tp2017187x2.tif]
